# Prevalence and determinants of post--abortion family planning utilization in a tertiary Hospital of Northwest Ethiopia: a cross sectional study

**DOI:** 10.1186/s40834-020-00143-4

**Published:** 2020-12-14

**Authors:** Enyew Abate, Yolanda R. Smith, Walelign Kindie, Addisu Girma, Yonas Girma

**Affiliations:** 1 College of Medicine and Health Sciences, Department of Obstetrics and Gynecology, Bahir Dar University, Bahir Dar, Ethiopia; 2 Department of Obstetrics and Gynecology, University of Michigan and Centers for International Reproductive Health Training (CIRHT), Ann Arbor, Michigan USA; 3 Debre Birhan Referral Hospital, Debre Birhan, Ethiopia; 4 Centers for International Reproductive Health Training (CIRHT), Addis Ababa, Ethiopia

**Keywords:** Post abortion family planning, Abortion services, Bahir Dar, Ethiopia

## Abstract

**Background:**

Provision of post abortion contraception following an abortion is an excellent opportunity to address unmet family planning needs of women. In Ethiopia, post abortion family planning is minimal and underutilized. The objective of this study is to assess determinant factors for utilization of contraception following any abortion process (induced and/or spontaneous) among reproductive age women (15–49 years) in a tertiary hospital of North West Ethiopia.

**Methods:**

A cross-sectional study was conducted on 423 clients who presented for either spontaneous or induced abortion care from September 2016 to August 2017 in Felege Hiwot referral hospital, North West Ethiopia. Respondents were identified using a consecutive sampling method. Data was collected in clinic using an interviewer administered pre-tested questionnaire administered after services were completed. Factors associated with use of post-abortion family planning were explored using multivariable logistic regression analysis.

**Results:**

64.8% of clients who presented for abortion care received family planning services before discharge from the hospital. Family planning counseling during service provision [AOR: 25.47, 95% CI: (9.11, 71.58)], having previous information about family planning [AOR: 2.16, 95% CI: (1.09, 4.23)], gestational age of index pregnancy less than 3 months [AOR: 1.78, 95% CI:(1.13, 3.05)], being a housewife [AOR: 0.32, 95% CI: (0.16, 0.65)] and monthly income > 5000 ETB [AOR: 0.38, 95% CI:(0.16,0.98)] are significantly associated with post abortion family planning utilization.

**Conclusions:**

The proportion of post abortion family planning utilization is good but could be improved. Education before and especially at the time of abortion services strongly influenced the usage of family planning services. The government and regional health bureau at large as well as health care providers at each health system level have an opportunity to provide information and counsel women on family planning methods to increase utilization of post abortion contraception.

## Background

The World Health Organization (WHO) estimates that abortion related complications are responsible for the deaths of approximately 800,000 women each year worldwide, which accounts for 19.6% of all maternal deaths [1, 2].

Ethiopia has one of the world’s highest maternal mortality ratios, 412 per 100,000 live births [3]. In Ethiopia, 38% of pregnancies are unintended, with an estimated 620,300 induced abortions performed each year. Among these pregnancy terminations, 53% occur in health facilities and 47% are performed outside of health facilities [4].

Family planning is a successful strategy to address this issue, and overall prevents 32% of maternal deaths from all causes [5]. Post abortion family planning is family planning that is provided to clients or individuals after a spontaneous or an induced abortion [6]. This post abortion period is a time when women are highly motivated to avoid or delay another pregnancy [7, 8]. This provides an opportunity to offer services immediately after the provision of abortion care.

Most unwanted pregnancies in developing countries occur as a result of restricted access to family planning services [9, 10]. In Ethiopia, 22% of women have an unmet need for family planning [3]. Providing voluntary post abortion family planning options generally reduces subsequent unintended pregnancies and further decreases maternal mortality [1, 6]. A consensus statement developed by Post Abortion Care Consortium and International Conference on Population and Development (ICPD) stated that the pregnancy-abortion-pregnancy cycle must be broken by providing post-abortion contraception to those who are in need of it [11].

Multiple studies done in Asia, South America and Africa on post-abortion family planning utilization showed wide variations in use, ranging from 61 to 97% [10, 12, 13]. A cross-sectional study performed in three health facility based settings in Ethiopia demonstrated that 48–59% of clients left their institution with post-abortion family planning services which showed wide variations in the percentage of women who received post-abortion contraception [14–16]. In addition, comparative studies from countries outside Ethiopia reveal a greater acceptance and use of modern contraceptives in women who received post abortion family planning counseling and services relative to the no-program group [17–19].

Several factors are associated with women’s decisions regarding starting post-abortion family planning. Studies done in Ethiopia indicate that urban residence, higher maternal education level, occupation, undergoing spontaneous abortion, receiving post abortion family planning counseling, good knowledge of family planning, and availability of family planning are significantly associated with uptake of family planning in the post-abortion period [16, 20–24].

Research performed around the world shows variation in the utilization of post-abortion family planning and type of methods offered and identified determinant factors. The majority of the research done in Ethiopia is community based and or health facility based studies.

No identified Ethiopian studies have sampled patients only from a tertiary teaching hospital to assess family planning utilization following abortion, where more complicated cases of abortion and women with advanced gestational age will come and where medical training occurs which may reflect what happens to the community after trainees leave.

Therefore, this study was done to provide information on the proportion and determinant factors for utilization of post abortion contraception in a tertiary teaching hospital in Ethiopia.

## Methods and materials

### Study setting, design and population

The study was an institutional cross-sectional study using quantitative data collection methods. It was conducted at Felege Hiwot referral hospital (FHRH), Bahir Dar, northwest Ethiopia from September 1, 2016 to August 31, 2017. FHRH is comprehensive specialized referral and teaching hospital found in Bahir Dar city, the capital of Amhara regional state which is located 563 Km away from the capital city Addis Ababa. It serves as referral hospital for South Gondar, East and West Gojjam, Awi zone and Benshangul Gumuz region providing service for about nine million populations.

We included all women who came for abortion care to get abortion services from FHRH. The sample size was calculated with the single population proportion formula: using 95% CL, 5% marginal error [25]. Using data on proportion of post-abortion family planning utilization done in Dessie, North East Ethiopia, which was 47.5% [15] and adding a 10% non-response rate, the planned sample size was 423.

### Data collection

Women were approached sequentially by trained bachelor degree holder nurses after their clinical visit was completed and while preparing to leave the hospital, until the desired sample size was achieved. Women who consented to participate completed a semi-structured interviewer administered questionnaire with 24 questions. The questionnaire was initially prepared in English and translated to Amharic language on a printout paper. The questions covered socio demographic characteristics, reproductive performance, previous abortion history, previous knowledge and utilization of any contraceptive methods, interest in future fertility, current pregnancy and abortion related circumstances which were obtained from the clients and health care provider information which was obtained from the abortion registration logbook.

### Data analysis

Data was entered and cleaned using Epi-data version 3.1, independently verified, exported and analysis was done using SPSS Version 22.0.

Descriptive statistics were used to assess the percentage of individual client characteristics and percentage of utilization of post-abortion family planning.

Bivariable analyses were performed, examining the association of selected independent variables with the outcome of post-abortion family planning utilization. Predictors with *p* values less than 0.25 were entered into subsequent multivariable models employing forward selection approach. The final multivariable logistic regression modelling was built by including predictors with p value less than 0.05 was undertaken to evaluate the net effects of set of predictor variables over the utilization of post abortion family planning.

## Results

### Sociodemographic characteristics

A total of 423 post abortion women participated in the study. Among the study participants, the majority of women were in the age group between 25 and 29 years (33.3%), resided in urban areas (58.9%), married (74%) and had access to at least one communication media (86.1%) (Table 1).

**Table 1:** Sociodemographic characteristics of study participants in Felege Hiwot referral hospital from Sep 2016- Aug 2017

Characteristics	Total	
	Number	Percent %)
**Age in years**		
Less than 19	62	14.7
20–24	111	26.2
25–29	141	33.3
30–34	63	14.9
> 35	46	10.9
**Place of residence**		
Urban	249	58.9
Rural	174	41.1
**Current marital status**		
Married	313	74
Not married	110	26
**Religion**		
Orthodox	361	85.3
Islam	57	13.5
Others	5	1.2
**Educational status**		
illiterate	162	38.3
Primary school	83	19.6
Secondary school	95	22.5
Higher education	83	19.6
**Occupation**		
Employed	117	27.7
Student	71	16.8
House wife	132	31.2
Daily laborer	27	6.4
Farmer	76	17.9
**Monthly income in birr**^a^		
< 5000	372	87.9
> 5000	51	12.1
**Availability of communication media**		
Yes	364	86.1
No	59	13.9

^a^One Ethiopian Birr is 0.027 United states Dollar as of September 22, 2020

### Obstetric and contraceptive history

Among the women participants, most of them had no child, had no previous abortion and wanted to be pregnant with in the next 6 months following the abortion process. Two thirds of women had spontaneous abortion and for majority abortion was done and/or completed by male nurses or midwives by using surgical methods. Most of the study participants had received counseling on family planning (Table 2).

**Table 2:** Description of obstetrics, contraceptive and abortion related factors of study participants in Felege Hiwot referral hospital from Sep 2016- Aug 2017

Characteristics	Total	
	Number	Percent (%)
**Number of alive children**		
None	217	51.3
One to four	170	40.2
> five	36	8.5
**Need for near future pregnancy**		
Yes	200	47.3
No	223	52.7
**Previous history of abortion**		
Yes	86	20.3
No	337	79.7
**Previous history of abortion care service**		
Yes	60	14.2
No	363	85.8
**Previous information about FP**		
Yes	345	81.6
No	78	18.4
**Currently counseled on FP**		
Yes	380	89.8
No	43	10.2
**Pregnancy planned**		
Yes (Planned)	225	53.2
No (Unplanned)	198	46.8
**Pregnancy wanted and supported**		
Yes (Wanted and supported)	290	68.6
No (Unwanted and unsupported)	133	31.4
**Gestational age of current pregnancy**		
Less than three months	185	43.7
Three to seven months	238	56.3
**Type of current abortion**		
Spontaneous	292	69
Induced	131	31
**Type of abortion procedure**		
Medication abortion	115	27.2
MVA	242	57.2
Both	66	15.6
**Sex of service provider**		
Male	349	82.5
Female	74	17.5
**Profession of service provider**		
Nurse or midwifery	279	66
General practitioner and above	144	34.1

### Utilization of post abortion contraception

Of all the study participants, nearly two third (274, 64.8%) had left the hospital after they have received family planning service. A little more than one third of women (35.2%) didn’t receive any type of family planning and among them,30% of women said nobody discussed the issue of contraception and another one third (30%) said they wanted to be pregnant in the near future.

Among women who received post abortion family planning services, majority (82.5%) had received upon discharge to their home and only 17.5% received services immediately after treatment. From the different contraceptives given to those women, Depo Provera injectable contraception was the most chosen method (138, 50%) and the least chosen method was traditional method (6, 2%).

### Factors associated with post abortion contraceptive utilization

The result of the Bivariate and Multivariable logistic regression analysis is displayed in Table 3. The Adjusted Odds Ratio (AOR) revealed that receiving family planning counseling during service provision (25.47[95% CI: 9.11, 71.58]), occupation of the patient (0.32[95% CI: 0.16,0.65]), having previous information about family planning (2.16 [95% CI: 1.09, 4.23]), gestational age of current pregnancy (1.78[95% CI: 1.13, 3.05]) and monthly income of the family (0.38[95% CI: 0.16,0.98]) have a significant impact on the utilization of post abortion family planning. Particularly, among Sociodemographic characteristics, women who are house wife were twenty times less likely to utilize PAFP. Similarly, the odds of using of PAFP in women whose monthly income was 5000 Ethiopian birr (ETB) and above were thirty-eight times less likely than their counter parts.

**Table 3:** Factors associated with utilization of post abortion contraception, logistic regression analysis, Felege Hiwot referral hospital, Ethiopia, from Sep 2016- Aug 2017

Variables	COR (95%)	AOR (95%)	AOR ***P***-value
**Occupation**			
Employed	1	1	.
House wife	1.10 (0.78, 1.54)	0.32 (0.16,0.65)	.002
**Monthly income in birr**			
< 5000	1	1	
> 5000	1.41 (0.51,2.31)	0.38 (0.16,0.98)	.037
**Previous information about FP**			
Yes	2.05 (1.64,2.57)	2.16 (1.09,4.23)	.028
No	1	1	
**Currently counseled on FP**			
Yes	2.59 (2.07,3.24)	25.47 (9.11,71.58)	<.001
No	1	1	
**Gestational age of current pregnancy**			
Less than three months	2.36 (1.72, 3.24)	1.78 (1.13, 3.05)	.038
Three to seven months	1	1	

The odds of utilization of post abortion family planning by women who received family planning counseling during service provision was 25.47 times than those women who didn’t. Likewise, the odds of using PAFP in women who have previous information about family planning was 2.16 times to those women who didn’t have previous information. Similarly, the odds of using PAFP in women whose current gestational age of pregnancy was less than 3 months was 1.78 times those whose gestational age was 3 to 7 months.

## Discussion

This cross-sectional study was conducted to assess the utilization of post-abortion family planning and associated factors in women who receive abortion care service. Accordingly, the proportion of use of post abortion family planning in this study was 64.8%. When compared with the studies done in Dessie (47.5%), Debre Markos (59.2%) and Gurage (56.5%), it is higher [14–16]. The possible explanations for these could be differences in study set up and time difference. In Dessie and Debre Markos studies, the study was performed in public, private and Non-Government Organization (NGO) clinics and in Gurage; they studied utilization in facilities at all levels of health system, whereas the current study was done in tertiary hospital. However, the studies done in Mexico (82%) and Brazil (97%), both had utilization above our study [10, 12]. This might be as result of different study design and set up. In Brazilian study they used a cohort study [10]. Another explanation might be excellent quality of service and health service coverage. On the other hand, our results are comparable with other studies done in Ghana (65%) and Burkina Faso (65.7%) [19, 26].

Client occupation is one factor which was identified to be associated with PAFP. On the contrary, in prior studies in Dessie [15] and Debre Markos [16] occupation had no statistically significant association with utilization of post abortion family planning. Our study found that women who are housewives were less likely to utilize PAFP. The possible reason could be that housewives have more time and support at home to raise a child, while women who are employed may intend to delay pregnancy.

Women whose monthly income was 5000 ETB and above were less likely to use PAFP than those women whose monthly income was less than 5000 ETB. This is contrary to another Ethiopian study from Addis Ababa, which found no significant association between use of post-abortion contraception and monthly income of the client [23].

Respondents who have previous information about family planning and those who were provided with post abortion family planning counseling were more likely to use PAFP than those who did not have previous information and were not counseled. These results are similar to those from other studies done in Debre Markos, Kenya and Turkey [1, 16]. This emphasizes the impact that post-abortion family planning counseling has on utilization of post abortion family planning.

Clients whose gestational age is less than 3 months in the current pregnancy were more likely to use post abortion family planning than those whose gestational age is 3 to 7 months. However, the studies from Dessie and Addis Ababa did not find a similar association [15, 20]. One possible explanation may be that our second trimester abortions have included women who had an induced abortion for medical reason following planned pregnancy. Therefore, these women may not wish to delay another pregnancy following the abortion process.

This was a single site study done in a referral hospital where generalization could be compromised as single hospital will not represent the community. The study also may not give the real utilization of post abortion family planning service because perhaps clients might show courtesy bias during the exit interview even if they were assured that all their words will be kept confidential.

## Conclusions and recommendations

In this study post-abortion contraceptive prevalence in a tertiary hospital was low as compared with studies done outside the country, even if it is higher than studies done in our country. Still there is more to be done to increase post-abortion contraception utilization. Family planning counseling during service provision, being a housewife, having previous information about family planning, monthly income and gestational age of index pregnancy are significantly associated with post-abortion family planning utilization. The type of abortion, miscarriage or induced, did not impact the interest and acceptance of contraception. We strongly recommend the government and regional health bureau at large and health care providers at each health system level in particular to provide family planning counseling for those women who come for any type of abortion related service.

## Data Availability

All generated data are included in the manuscript.

## Abbreviations

AIDS: Acquired Immune Deficiency Syndrome
ARHB: Amhara Regional Health Bureau
CIRHT: Centers for International Reproductive Health Training
EDHS: Ethiopian Demographic Health Survey
ETB: Ethiopian birr
FHRH: Felege Hiwot Referral Hospital
HIV: Human Immune Virus
ICPD: International Conference on Population and Development
IUCD: Intra Uterine Contraceptive Device
MVA: Manual Vacuum Aspiration
PAFP: Post Abortion Family Planning
WHO: World Health Organization
